# Sex differences in urea breath test results for the diagnosis of *Helicobacter pylori* infection: a large cross-sectional study

**DOI:** 10.1186/s13293-017-0161-7

**Published:** 2018-01-02

**Authors:** Ido Eisdorfer, Varda Shalev, Sophy Goren, Gabriel Chodick, Khitam Muhsen

**Affiliations:** 1 Department of Epidemiology and Preventive Medicine, School of Public Health, Sackler Faculty of Medicine, Tel Aviv University, Ramat Aviv, 69978 Tel Aviv, Israel; 2 Medical Informatics Division, Maccabi Health Services, Tel Aviv, Israel

**Keywords:** *H*. *pylori* infection, Urea breath test, Sex differences, Smoking

## Abstract

**Background:**

*Helicobacter pylori* causes peptic ulcer disease and gastric cancer only in a subset of infected persons. Sex differences were shown in results of urea breath test (UBT), a commonly used test for the diagnosis of *H*. *pylori* infection. However, factors that might explain these differences, or affect UBT values, are not fully understood. We examined differences in UBT values between *H*. *pylori*-infected men and women while adjusting for background characteristics such as age, body mass index (BMI), and smoking.

**Methods:**

A cross-sectional study was undertaken using coded data from the computerized database of Maccabi Health Services in Israel. Included were adults examined for UBT during 2002–2012 and were found *H*. *pylori* positive (UBT > 3.5‰). Multivariable linear mixed models were performed to assess the relationship between sex and UBT quantitative results, while adjusting for background characteristics.

**Results:**

A total of 76,403 patients were included (52% of examined patients during the study period). Adjusted mean UBT value was significantly higher in women 33.8‰ (95% CI 33.4, 34.1) than in men 24.9‰ (95% CI 24.5, 25.3). A significant (*P* < 0.001) interaction was found between sex and smoking, showing diminished sex-differences in UBT results in smokers. Adjusted mean UBT values increased significantly with age and decreased with BMI, and it was higher in people who lived in low vs high socioeconomic status communities and lower in smokers vs non-smokers.

**Conclusions:**

Systemic differences exist between men and women in quantitative UBT results. Host-related and environmental factors might affect UBT quantitative results. These findings have clinical implications regarding confirmation of the success of *H*. *pylori* eradication after treatment in various subgroups.

## Background

*Helicobacter pylori* colonizes the gastric mucosa and causes chronic gastritis. Most *H*. *pylori*-infected persons remain asymptomatic, but some might develop peptic ulcer disease and gastric cancer [1–4]. Men have higher risk for peptic ulcers [5] and gastric cancer [6, 7] compared to women, although the difference between sexes in *H*. *pylori* infection prevalence is small [8].

*H*. *pylori* invasive detection methods are based on gastric biopsy and include culture, histology, and rapid urease test (reviewed in [9, 10]). Among the non-invasive tests, urea breath test (UBT) is highly accurate [11]; it is recommended for the diagnosis of *H*. *pylori* in test-and-treat strategy and for the confirmation of *H*. *pylori* eradication [12].

UBT is based on urease activity of *H*. *pylori* and its ability to hydrolyze orally ingested isotopically labeled urea (^13^C or ^14^C urea) into ammonia and labeled carbon dioxide (CO_2_), which is eventually exerted through the lungs in exhaled breath [10, 13]. The amount of labeled carbon dioxide is measured as ^13^C to ^12^C ratio and expressed as delta over baseline (DOB) value. Typically, DOB of 3.5‰ or greater is employed to determine the presence of *H*. *pylori* infection, and some use a cutoff value of 5‰ [10]. DOB values were shown to be positively correlated with gastric *H*. *pylori* bacterial load or density and severity of gastritis [14–18].

Some studies reported significantly higher DOB values in *H*. *pylori*-infected females compared to infected males [19–22]; however, the explanation of these observations is not fully known. The aims of the current study were to examine differences in UBT results between *H*. *pylori*-infected men and women and to examine whether these differences might be explained or modified by sociodemographic and clinical characteristics, such as age, body mass index (BMI), and smoking.

## Methods

### Study design and population

A cross-sectional study was undertaken using anonymous data retrieved from the computerized database of Maccabi Health Services (MHS) Health Maintenance Organization (HMO). MHS is the second largest HMO in Israel with two million insured persons (~ 25% of the Israeli population). The source population comprised adults aged ≥ 25 years who performed the ^13^C-UBT between 2002 and 2012. This referral sample included mainly persons with symptoms and complaints consistent with clinical indications for the diagnosis of *H*. *pylori* infection. Exclusion criteria were anti-*H*. *pylori* eradication therapy (according to purchases of medications) or proton pump inhibitors 4 weeks before the UBT, history of bariatric surgery, a prior diagnosis of gastric cancer, and diagnosis of other cancers within 2 years from the UBT. Information on cancer was obtained via linkage with Israel’s National Cancer Registry.

### Data extraction and definitions

Information was obtained on UBT result, birth date, sex, town of residence, and smoking (ever, never, and unknown). Age at the first UBT was grouped into five categories: 25–34, 35–44, 45–54, and 65–95 years. Socioeconomic status (SES) was defined based on the socioeconomic rank of place of residence at the level of town, as defined by the Israel Central Bureau of Statistics [23]. The ranks are on a scale from 1 to 10, with higher ranks representing a higher socioeconomic status. This aggregative socioeconomic index reflects a combination of basic characteristics of a specific geographical unit investigated, mainly financial resources of the residents, housing conditions, motorization level, education, and employment [23]. Communities with socioeconomic ranks of 1–5 and 6–10 were classified as low and intermediate/high, respectively. BMI (weight in kilograms (kg)/height^2^ in meters (m)) was categorized into the following categories: < 18.5, 18.5–24.9, 25–29.9, and ≥ 30 kg/m^2^.

Patients with a UBT result of DOB > 3.5‰ were considered *H*. *pylori* positive. If more than one UBT was performed, we used the first test. The presence of peptic ulcer disease was defined based on the International Classification of Diseases, 9th revision (ICD-9) and corresponding internal codes at MHS.

### Statistical analysis

Student’s *t* test was used to examine differences in mean UBT values among infected persons according to sex, and one-way analysis of variance (ANOVA) was used to examine differences in UBT values according to age, BMI categories, SES, and smoking. Multivariable analyses were performed using mixed linear models to assess the relationship between sex and UBT values while controlling for other independent variables; interactions between sex and other independent variables were tested in these models. Pooled and sex-stratified analyses were performed. Two-tailed *P* < 0.05 was considered significant. Data were analyzed using SPSS version 23 (IBM, Armonk, New York, USA).

### Ethical approval

The study protocol was approved by the Helsinki committee of Assuta Medical Center and the ethics committee of Tel Aviv University. Since this was a retrospective study in which we used coded (anonymized) administrative data from electronic medical records, an exemption from informed consent was given by the Helsinki committee.

## Results

During the study period, 146,864 persons (60.7% females), with a mean age of 42.7 years (standard deviation (SD) 12.7), were referred to UBT and met the inclusion criteria. The mean age of men and women was 42.8 (SD 12.5) and 42.7 (SD 12.8), respectively, *P* = 0.6. *H*. *pylori* infection (UBT > 3.5‰) was evident in 76,403 persons (52.0%), and it was 52.9 and 51.5% in men and women, respectively. Men had a higher mean BMI than women, 26.6 kg/m^2^ (SD 4.1) vs 25.5 kg/m^2^ (SD 5.1), *P* < 0.001, and were smokers more often compared to women, 19.5 vs 10.7%, *P* < 0.001. The distribution of SES was similar between men and women: 41.0, 26.3, and 26.3% of the men lived in low, intermediate, and high SES towns, respectively, vs 40.5, 26.6, and 26.5% of the women.

### Mean values of UBT results according to background characteristics

Among *H*. *pylori*-infected persons, UTB results ranged from 3.51 to 175.0‰ with a mean DOB value of 30.2‰ (SD 20.9) and a median of 24.6‰ (interquartile range [IQR 24.8]). The mean UBT values were significantly higher in women than in men, 34.6‰ (SD 22.8) and 23.6‰ (SD 15.4), respectively, with a mean difference of 11.0‰ (95% CI 10.7, 11.3); *P* < 0.001. The median DOB value was 28.8‰ (IQR 28.6) and 19.6‰ (IQR 18.1) in women and men, respectively.

Overall, the mean UBT values increased significantly (*P* < 0.001) with age by 3.7 units from 28.6‰ (SD 20.3) in the youngest age group to 32.3‰ (SD 22.4) in the oldest age group. Mean UBT values decreased as BMI increased (*P* < 0.001) by 9 units, from 36.5‰ (SD 25.4) in persons with BMI < 18.5 kg/m^2^ to 27.2‰ (SD 18.1) in obese persons with BMI ≥ 30 kg/m^2^. Smokers had a lower mean UBT value compared to non-smokers, with a mean difference of 8.1 (95% CI 7.7, 8.5) DOB units (*P* < 0.001) (Table 1).

**Table 1:** Unadjusted mean DOB of ^13^C-UBT values of *H*. *pylori*-infected patients according to baseline characteristics

	Overall			Men			Women			
	*N*	Mean UBT value (SD), ‰	*P*	*N*	Mean UBT value (SD), ‰	*P*	*N*	Mean UBT value (SD), ‰	*P*	*P* for interaction by sex
Sex										
Men	30,543	23.6 (15.4)	< 0.001^a^	–	–		–	–		
Women	45,860	34.6 (22.8)		–	–		–	–		
Age, years^b^		df = 4, *F* = 52.8	< 0.001^b^		df = 4, *F* = 60.7	< 0.001^c^		df = 4, *F* = 29.6	< 0.001^b^	< 0.001^c1^
25–34	22,919	28.6 (20.3)		9280	22.0 (14.6)		13,639	33.1 (22.3)		
35–44	25,643	30.8 (21.3)		10,478	23.4 (15.0)		15,165	35.9 (23.4)		
45–54	15,768	30.4 (20.6)		6166	24.4 (15.4)		9602	34.3 (22.5)		
55–64	7942	31.0 (20.6)		2982	25.3 (16.4)		4960	34.5 (22.0)		
65–95	4131	32.3 (22.4)		1637	27.2 (18.9)		2494	35.7 (23.8)		
SES of place of residence		df = 2, *F* = 45.1	< 0.001^b^		df = 2, *F* = 4.8	0.008^c^		df = 2, *F* = 54.4	< 0.001^b^	< 0.001^c2^
1–5 (low SES)	35,358	30.9 (21.3)		14,272	23.8 (15.5)		21,806	35.8 (23.2)		
6–10 (intermediate–high SES)	36,549	29.6 (20.6)		14,474	23.5 (15.4)		22,075	33.7 (22.4)		
Missing	4496	28.8 (20.0)		1797	22.6 (14.8)		2699	32.9 (21.8)		
BMI, kg/m^2^		df = 4, *F* = 268.3	< 0.001^b^		df = 4, *F* = 65.1	< 0.001^c^		df = 4, *F* = 89.4	< 0.001^b^	0.031^c3^
< 18.5	1871	36.5 (25.4)		283	27.7(18.9)		1588	38.0 (26.1)		
18.5–24.9	30,933	32.6 (22.4)		9906	25.2 (16.4)		21,027	36.2 (24.0)		
25.0–29.9	26,100	28.8 (19.7)		13,011	23.4 (15.2)		13,089	34.2 (22.0)		
≥ 30	14,140	27.2 (18.1)		5648	21.3 (13.6)		8492	31.0 (19.6)		
Missing	3359	27.7 (20.0)		1695	22.2 (14.9)		1664	33.2 (22.8)		
Smoking		df = 2, *F* = 716.6	< 0.001^b^		df = 2, *F* = 217.8	< 0.001^c^		df = 2, *F* = 269.7	< 0.001^b^	< 0.001^c4^
Ever	11,862	23.7 (16.9)		6658	20.3 (14.4)		5204	28.0 (19.6)		
Never	43,754	31.7 (21.4)		16,717	24.9 (16.0)		27,037	36.0 (23.1)		
Unknown	20,787	30.6 (21.2)		7168	23.5 (15.3)		13,619	34.4 (22.9)		

*H*. *pylori* infection was defined as delta ^13^C-UBT of > 3.5 per thousand, see text for details

*BMI* body mass index, *df* degrees of freedom, *DOB* delta over baseline, *SD* standard deviation, *UBT* urea breath test, *SES* socioeconomic status

^a^*P* value by Student’s *t* test; *t* = − 74.0, df = 76,385.8

^b^*P* value by ANOVA

^c^*P* values from linear mixed model assessing interaction between sex and each independent variable in separate models: ^1^df = 4, *F* = 19.5; ^2^df = 2, *F* = 18.8; ^3^df = 4, *F* = 2.6; and ^4^df = 2, *F* = 32.3

A stratified analysis by sex showed that in men, the average UBT values increased by 5 units from 22.0‰ (SD 14.6) at the youngest age group 25–34 to 27.2‰ (SD 18.9) at age 65–95 years, while in women, the corresponding increase was 2.6 units from 33.1‰ (22.3) to 35.7‰ (23.8). The difference in UBT values between persons living in low vs intermediate/high SES communities was evident in women (2.1 units) more than in men (0.3 units). The difference according to BMI was found in both sexes, with significant interaction (*P* = 0.031), suggesting a reduced difference between sexes in obese persons. Differences in the mean UBT values according to smoking were also observed in both sexes, but among smokers, the gap in UBT values between men and women was reduced by 3 units (*P* < 0.001 for interaction) (Table 1).

In a pooled multivariable analysis that adjusted for baseline characteristics, *H*. *pylori*-infected women still had significantly (*P* < 0.001) higher mean UBT values than men, although the difference (9.7‰ [95% CI 9.0, 10.4]) in DOB units was reduced compared to unadjusted results. Additionally, the differences according to age groups, SES of place of residence, BMI, and smoking remained significant (*P* < 0.001) (Table 2).

**Table 2:** Adjusted UBT values per thousand of *H*. *pylori*-infected patients according to baseline characteristics

	Estimate (95% CI)*	Adjusted mean UBT value (95% CI)
Intercept	27.6 (26.5, 28.6)	Overall 29.3 (29.0, 29.6)
Sex^a^		
Men	0 (reference)	24.9 (24.5, 25.3)
Women	9.7 (9.0, 10.4)	33.8 (33.4, 34.1)
Age, years^b^		
25–34	0 (reference)	26.4 (26.1, 26.8)
35–44	2.1 (1.5, 2.7)	29.2 (28.8, 29.6)
45–54	3.4 (2.7, 4.0)	29.4 (29.0, 29.8)
55–64	4.2 (3.3, 5.0)	30.1 (29.5, 30.6)
65–95	5.8 (4.7, 6.9)	31.6 (30.9, 32.3)
SES of place of residence^c^		
1–5 (low SES)	0.8 (0.3, 1.3)	30.2 (29.8, 30.4)
6–10 (intermediate–high SES)	0 (reference)	28.5 (28.1, 28.8)
BMI kg/m^2d^		
< 18.5	0 (reference)	33.5 (32.6, 34.5)
18.5–24.9	− 2.8 (− 3.8, − 1.8)	30.7 (30.5, 31.0)
25.0–29.9	− 5.5 (− 6.4, − 4.5)	28.1 (27.8, 28.4)
≥ 30	− 8.6 (− 9.6, − 7.6)	24.9 (24.6, 25.3)
Smoking^e^		
Unknown	− 1.3 (− 1.9, − 0.7)	30.5 (30.1, 30.9)
Ever	− 4.6 (− 5.2, −4.0)	25.6 (25.1, 26.0)
Never	0 (reference)	31.9 (31.6, 32.3)

*BMI* body mass index, *CI* confidence intervals, *DOB* delta over baseline, *UBT* urea breath test, *SES* socioeconomic status

*P < 0.001 for all variables was obtained in type III tests of fixed effects. Denominators: degree of freedom (df) of all variables was 68,699. Numerators: ^a^df = 1, *F* = 1685.2; ^b^df = 4, *F* = 91.1, ^c^df = 2, *F* = 122.2; ^d^df = 4, *F* = 276.7; ^e^df = 2, *F* = 414.7

This model showed interactions (*P* < 0.001) between sex and other independent variables. Overall, women had a higher adjusted mean DOB than men in about 9.7 units; in women aged 35–44, the difference was increased by 1.3 units (*P* = 0.001), and in men aged 45–54, 55–64, and 65–95, the difference was reduced by 0.8 (*P* = 0.059), 1.1 (*P* = 0.045), and 1.3 (*P* = 0.074) units, respectively. In women who lived in low SES towns, the difference in UBT values increased by 1.8 units (*P* < 0.001). The difference between men and women in UBT values was reduced by 3.5 units (*P* < 0.001) in smokers.

Multivariable analyses, conducted separately for men and women, showed that age, SES, BMI, and smoking were significant determinants of UBT results in both sexes (Table 3).

**Table 3:** Adjusted UBT values of *H*. *pylori*-infected patients according to baseline characteristics by sex

	Estimate (95% CI)^a^	Estimate (95% CI)^b^	Adjusted mean UBT value (95% CI of the mean)	
	Men	Women	Men	Women
Intercept	27.6 (25.9, 29.5)	37.2 (36.0, 38.4)	Overall 24.9 (24.4, 25.4)	Overall 33.7 (33.3, 34.2)
Age, years				
25–34	0 (reference)	0 (reference)	22.0 (21.4, 22.5)	30.9 (30.4, 31.4)
35–44	2.0 (1.5, 2.4)	3.5 (2.9, 4.0)	24.0 (23.4, 24.5)	34.4 (33.9, 34.9)
45–54	3.2 (2.7, 3.7)	2.6 (2.0, 3.3)	25.2 (24.6, 25.8)	33.6 (33.0, 34.1)
55–64	4.0 (3.3, 4.6)	3.2 (2.5, 4.0)	26.0 (25.2, 26.7)	34.2 (33.4, 34.9)
65–95	5.6 (4.8, 6.4)	4.7 (3.7, 5.7)	27.6 (26.7, 28.5)	35.6 (34.7, 36.6)
SES of place of residence				
1–5 (low SES)	0.8 (0.4, 1.1)	2.7 (2.3, 3.2)	25.3 (24.8, 25.9)	35.1 (34.6, 35.6)
6–10 (intermediate–high SES)	0 (reference)	0 (reference)	24.6 (24.0, 25.1)	32.4 (31.9, 32.8)
BMI, kg/m^2^				
< 18.5	0 (reference)	0 (reference)	29.1 (27.2, 30.9)	38.1 (36.9, 39.3)
18.5–24.9	− 3.2 (− 5.0, − 1.4)	− 2.6 (− 3.8, − 1.4)	25.8 (25.5, 26.2)	35.4 (35.0, 35.8)
25.0–29.9	− 5.5 (− 7.3, − 3.7)	− 5.4 (− 6.7, − 4.2)	23.5 (23.2, 23.9)	32.6 (32.2, 33.1)
30+	− 7.7 (− 9.6, − 5.9)	− 9.2 (− 10.5, − 7.9)	21.3 (20.9, 21.8)	28.8 (28.3, 29.4)
Smoking				
Unknown	− 1.3 (− 1.7, − 0.8)	− 1.7 (− 2.2, − 1.2)	25.6 (25.0, 26.2)	35.3 (34.8, 35.8)
Ever	− 4.6 (− 5.1, − 4.1)	− 8.1 (− 8.8, − 7.4)	22.3 (21.7, 22.9)	28.9 (28.2, 29.6)
Never	0 (reference)	0 (reference)	26.9 (26.4, 27.4)	37.0 (36.6, 37.4)

*BMI* body mass index, *DOB* delta over baseline, *SD* standard deviation, *UBT* urea breath test, *SES* socioeconomic status

^a^*P* < 0.001 for all variables was obtained in type III tests of fixed effects. Denominators: degree of freedom (df) of all variables was 27,123. Numerators: df = 1 (*F* = 16.7), 2 (*F* = 192.9), 3 (*F* = 109.2), and 4 (*F* = 73.6) for SES of place of residence, smoking, BMI, and age, respectively

^b^*P* < 0.001 for all variables was obtained in type III tests of fixed effects. Denominators: df of all variables was 41,573. Numerators: df = 1 (*F* = 148.2), 2 (*F* = 259.6), 3 (*F* = 173.4), and 4 (*F* = 47.2) for SES of place of residence, smoking, BMI, and age, respectively

### Secondary analysis

The mean values of UBT in uninfected men and women were similar, 0.71 (SD 0.60) and 0.76 (SD 0.56), respectively.

## Discussion

We examined sex differences in *H*. *pylori* infection prevalence and values of UBT in positive persons, in a large referral sample of adults. The prevalence of *H*. *pylori* infection in this sample was similar in men and women, 52.9 vs 51.5%. Among *H*. *pylori*-infected individuals, the mean delta UBT value was significantly higher in women than in men, 34.6 and 23.6‰, respectively. This difference was slightly attenuated after adjustment for background characteristics, 33.8 and 24.9‰ in women and men, respectively, with adjusted mean difference of 9.7‰ (95% CI 9.0, 10.4).

UBT measures the amount of labeled CO_2_ exerted through the lungs in exhaled breath [10, 13]. Thus, beyond the presence of *H*. *pylori* urease that hydrolyzes the urea into ammonia and labeled CO_2_, factors affecting endogenous production of CO_2_ have an impact on UBT quantitative results. Endogenous production of CO_2_ depends much on basal metabolic rate, which differs according to sex and body surface area (a function of weight and height).

Although we adjusted for host-related and environmental factors including age, SES, smoking, and BMI, *H*. *pylori*-infected women still had higher UBT values compared to men, thus suggesting that these background characteristics account only partially for the sex differences in UBT values and that there must be additional contributing factors. Variation between men and women was shown in intragastric acidity and plasma gastrin concentration profiles [24]. Urease activity was shown to be increased in more acidic conditions [25–27]; therefore, sex differences in intragastric pH might contribute to sex differences in DOB values. Studies of Mongolian gerbils have shown that gastric mucosal cytokine and epithelial cell response to *H*. *pylori* infection differ between males and females, with skewed cytokine response in females towards T-helper 1 profile [28], in addition to difference in the magnitude of anti-*H*. *pylori* gastric cytokine responses [28]. Collectively, these observations support the possibility of the involvement of sex-specific immunological or hormonal factors on UBT values. Hormonal factors might affect gastric mucosal blood flow and thickness of mucus layer [29]; the role of these factors in variation in UBT values warrants exploration.

Previous studies on sex differences in UBT values among *H*. *pylori*-infected people have also showed higher mean values of about 5 to 10 units in females compared to males [19–22]. However, adjustment for background characteristics beyond age was limited in previous studies. Interestingly, in both *H*. *pylori*-infected men and women, the mean UBT values increased with age, but it decreased with increased BMI. Smokers had a significantly lower mean UBT result than non-smokers, as well as persons who lived in high/intermediate SES towns compared to those who lived in low SES towns.

It was proposed that the magnitude of DOB of UBT might serve as an indicator for the severity of gastric inflammation and *H*. *pylori* bacterial load in the stomach [14–18, 30]. Values of DOB reflect also urease enzyme activity in various gastric pH levels. It was shown that *H*. *pylori* does not survive at either acidic or alkaline environments [25, 27]. Intragastric pH plays an important role in the activity of urease of *H*. *pylori*; acidic conditions increase urease activity and the bacterium’s survival [25–27]. Intragastric pH increases with the development of gastric atrophy [31]. Therefore, in the case of gastric atrophy, which develops with increased age, a decrease in DOB values would be expected. The increase in the severity of gastritis with age might explain the rise in DOB values with age, i.e., reflecting a greater gastric inflammation and activity of urease. The same might be applied to persons living in low SES communities, who usually acquire the infection at younger age compared to persons living in high SES communities [32, 33]. An additional or alternative explanation is that basal metabolic rate decreases with age [34, 35], thus leading the higher UBT values in older patients.

The absolute difference in UBT results in smokers and non-smokers infected with *H*. *pylori* was relatively large. The lower mean DOB values observed in smokers compared to non-smokers herein and by others [36] might be due to the harmful effects of smoking on gastric mucosa [37] and development of atrophic gastritis. Smoking is a known risk factor for peptic disease [36], atrophic gastritis [38], and gastric cancer [39]. Therefore, these findings suggest that interpretation of UBT results in smokers warrants attention.

The negative association of BMI with UBT values in both *H*. *pylori*-infected men and women is likely due to the differences in metabolic rate according to body size. *H*. *pylori* infection was shown to be associated with lower mean height [40]; thus, indirectly, it affects endogenous CO_2_ production.

*H*. *pylori* infection prevalence was lower in patients who lived in intermediate/high SES towns (47%) than those who lived in low SES towns (59%), *P* < 0.001, in agreement with previous reports [41, 42]. Surprisingly, we found that SES of place of residence was also inversely related to UBT quantitative results. This suggests high gastric *H*. *pylori* bacterial load in persons who live in low SES communities due to more intense exposure to the infection. Since we employed an aggregative SES index of place of residence, our observation might indicate possible influence of environmental factors on UBT results, including air pollution that affects lung function [43, 44].

The clinical importance of higher UBT results in *H*. *pylori*-infected women than men is not fully clear. One might expect a higher disease risk in groups displaying greater DOB values. However, men are at greater risk for peptic ulcer disease than women [36]. However, women suffer more often from functional dyspepsia [45, 46]. The recent Maastricht V/Florence Consensus Report on the management and treatment of *H*. *pylori* indicated that *H*. *pylori* gastritis is a distinct cause of dyspepsia and thus is considered an organic disease [12], whereas Rome III consensus considered *H*. *pylori*-related dyspepsia as functional. The question of whether the sex differences in dyspepsia might be related to higher UBT values in women remains to be elucidated. Some studies have reported a higher rate of *H*. *pylori* eradication failures in women than in men [47, 48], while the opposite was shown in another study [49]. Moreover, higher UBT values prior to the administration of anti-*H*. *pylori* therapy are associated with increased likelihood of treatment failure [49]. A clinical trial that assessed the effect of cranberry juice on *H*. *pylori* eradication in patients who received triple therapy showed significantly higher eradication rates in women who received both cranberry juice and triple therapy compared to placebo beverage and triple therapy [50]. Such effect was not observed in men [50]. Collectively, these findings demonstrate the clinical significance of UBT quantitative results.

Our study has some drawbacks. We used data from a large HMO database, routinely collected for patients’ clinical care. Data collecting methods on variables such as smoking and BMI may differ among medical staff members. Missing information on smoking status was evident in 27% of the study sample. In persons with *H*. *pylori* infection whose smoking status was unknown, the mean UBT values were similar to never smokers. Missing data on SES and BMI is low and seems quite random with low threat of bias.

Our study also has strengths. First is the use of a large population-based sample. Second is the employment of standard criteria for the classification of sociodemographic factors and *H*. *pylori* infection. Third, the UBT was performed in one laboratory throughout the study period. Lastly, our sample likely represents symptomatic persons, since only physicians can refer patients to UBT.

## Conclusion

In summary, in a large referral sample, we demonstrated systemic differences between men and women in quantitative UBT results. In addition, host-related and environmental factors such as age, SES of place of residence, BMI, and smoking affect UBT quantitative results in *H*. *pylori*-infected people. Hence, inference from UBT quantitative results on *H*. *pylori* gastric bacterial load and severity of gastritis should be made with caution. Clinical implications of these findings relate to confirmation of *H*. *pylori* eradication success or failure following therapy in various subgroups, and our observations raise a question of whether cutoff values for UBT should be sex-specific.
